# Trazodone induced euprolactinemic galactorrhea – a case report

**DOI:** 10.1192/j.eurpsy.2023.434

**Published:** 2023-07-19

**Authors:** D. Mortágua, D. Rafaela

**Affiliations:** ^1^Department of Psychiatry, Centro Hospitalar e Universitário de Coimbra; ^2^Institute of Medical Psychology, University of Coimbra, Coimbra, Portugal

## Abstract

**Introduction:**

Trazodone is an antidepressant that exerts its effect through serotonin reuptake inhibition and 5-HT2A and 5-HT2C receptor antagonism. Galactorrhea, as well as the increase in prolactin levels, have been seldom related to antidepressants. These adverse effects are more frequently observed with antipsychotic medication.

**Objectives:**

To present and discuss a case of Trazodone induced galactorrhea in a 24-year-old female patient diagnosed with a moderate depressive episode, without psychotic symptoms.

**Methods:**

Clinical case description and literature review.

**Results:**

We present the case of a healthy 24-year-old woman, medicated with oral contraceptives, presented to a Psychiatry Consultation due to worsening depressive and anxious symptoms. Prolonged-release Trazodone was initiated with the indication to gradually titrate up to 300 mg/day. On the third day of treatment (at the time, at a dose of 75 mg/day), the patient began to experience breast pain and galactorrhea. On the seventh day, due to continuation of the complaints, she went to a Gynecology Consultation, having carried out an analytical study, in which a prolactinemia value was registered within the normal range (18 ng/mL). The possibility of pregnancy or continued intake of anabolizing steroids was excluded. The condition reversed upon discontinuation of the drug.

**Conclusions:**

The endocrine and reproductive effects of antidepressants are uncommon and galactorrhea is only rarely mentioned as a possible adverse effect of this type of medication. The neurobiological mechanisms underlying this association are unclear. The existing literature points to the possibility that serotonergic antidepressants act by suppressing dopamine neurotransmission (by indirect inhibition of the tuberoinfundibular pathway), facilitating the release of prolactin and thus contributing to the increase in its levels. However, there are also case reports of antidepressant-induced galactorrhea in the presence of normal prolactin levels. In the present case, a state of euprolactinaemia was, in fact, verified. The findings reinforce the importance of carrying out more studies and on a larger scale, to better clarify the mechanisms underlying this association.

**Disclosure of Interest:**

None Declared

